# The value of interleukin-6 in predicting acute appendicitis in children and distinguishing complicated appendicitis: a systematic review and meta-analysis

**DOI:** 10.3389/fimmu.2026.1790229

**Published:** 2026-04-17

**Authors:** Longtao Tan, Cong Bo

**Affiliations:** 1 Department of Pediatric Surgery, Binzhou People’s Hospital, Binzhou, China; 2 Department of Cardiac Surgery, Binzhou People’s Hospital, Binzhou, China

**Keywords:** appendicitis, children, complicated appendicitis, interleukin 6, uncomplicated appendicitis

## Abstract

**Objectives:**

The identification of new, easily measurable biomarkers may assist clinicians in diagnosing and managing acute appendicitis. Although inflammatory markers have been applied in pediatric appendicitis diagnosis, the diagnostic and disease assessment value of interleukin-6 (IL-6) as a key inflammatory factor in this condition has not yet been systematically evaluated.

**Methods:**

We conducted a systematic search of electronic databases to identify all studies reporting IL-6 in children with a clinical diagnosis of acute appendicitis. We considered two comparisons: 1) appendicitis versus no appendicitis and 2) uncomplicated appendicitis (UA) versus complicated appendicitis (CA). We converted and analyzed the odds ratios from the multivariate analysis to determine the independent predictive value of IL-6 in pediatric acute appendicitis.

**Results:**

Seventeen studies were included in this meta-analysis. These studies enrolled a total of 3,104 patients. The IL-6 levels were significantly higher in patients with acute appendicitis (MD −19.99; 95% CI: −31.19 to −8.79; p = 0.0008; I^2^ = 73.8%). The levels of IL-6 were statistically significantly higher in the CA group (MD −95.75; 95% CI: −151.86 to −39.65; p < 0.0001; I^2^ = 86%). IL-6 may not be an independent predictor of CA in children (OR: 0.94; 95% CI: 0.75 to 1.18; I^2^ = 86%). Subgroup analysis and sensitivity analysis suggested that the diagnostic performance of IL-6 was not significantly modified by the detection method.

**Conclusion:**

IL-6 may serve as one of the markers for distinguishing UA from CA, although no clear association was found regarding its independent predictive value. Future prospective studies with larger sample sizes are warranted to establish higher-level evidence.

**Systematic Review Registration:**

https://www.crd.york.ac.uk/prospero/, identifier PROSPERO CRD420251234189.

## 1 Introduction

Acute appendicitis is the most common acute abdominal condition in children, and its timely and accurate diagnosis remains a core challenge in pediatric surgical practice. Among children presenting to the emergency department with acute abdominal pain, acute appendicitis accounts for 1%–8% of cases (1). Appendicitis is traditionally managed surgically with appendectomy. Children with uncomplicated appendicitis (UA) may be suitable for conservative management rather than surgical intervention (2–4). In contrast, those presenting with complicated appendicitis (CA) often require early surgery due to the consideration of poorer prognosis, increased healthcare costs (5), a sixfold higher readmission rate, and prolonged hospital stay associated with this condition (6).

Currently, the diagnosis of acute appendicitis is primarily based on clinical assessment, supplemented by imaging studies such as ultrasonography and conventional inflammatory markers, including white blood cell count (WBC) and C-reactive protein (CRP). However, each of these methods has limitations. For instance, while WBC and CRP are widely used, they lack specificity in the early stages of the disease or exhibit insufficient sensitivity in UA, making it difficult to distinguish between UA and CA. Therefore, there is an urgent clinical need to explore a more sensitive and specific biomarker to aid in early differential diagnosis and risk assessment.

Following bacterial invasion of the appendix wall, antigen-bound IgE binds to high-affinity receptors. This interaction triggers eosinophil infiltration and mast cell degranulation, leading to the release of inflammatory mediators. Ultimately, this cascade induces a cytokine-mediated inflammatory response characterized by Type I hypersensitivity (7–9). Interleukin-6 (IL-6), as a key early pro-inflammatory cytokine, is rapidly released by activated macrophages and T cells during acute inflammatory responses. Its serum concentration rises earlier than CRP and is considered a more direct indicator reflecting the severity of tissue damage and infection. In most studies, the levels of serum total bilirubin, IL-6, WBC, CRP, and procalcitonin (PCT) were consistently higher in children with CA compared to those with UA (10–12).

However, existing studies on the relationship between acute appendicitis in children and IL-6 have exhibited significant heterogeneity in evidence and inconclusive findings. Differences exist across studies in detection methods, cutoff value settings, study populations, and definitions of CA. Currently, there is a lack of a comprehensive, systematic quantitative review to synthesize this evidence, particularly one specifically addressing the following three progressive levels of analysis: 1) the overall discriminatory ability of IL-6 in distinguishing appendicitis from non-appendicitis conditions in children, 2) the role of IL-6 in differentiating UA from CA, and 3) the value of IL-6 as an independent predictor of complicated appendicitis.

We conducted a systematic review and meta-analysis to evaluate the predictive value of IL-6 in pediatric patients with acute appendicitis. Our primary objectives were to assess its ability to predict acute appendicitis, distinguish CA, and independently predict complicated appendicitis.

## 2 Materials and methods

A systematic literature review was conducted according to the Preferred Reporting Items for Systematic Reviews and Meta-Analyses (PRISMA) 2020 guideline (13). A comprehensive systematic literature search was performed across the following databases: PubMed, Embase, Cochrane, and Web of Science. The study protocol was prospectively registered in the PROSPERO database (registration number: CRD420251234189).

### 2.1 Literature search strategy

PubMed, Embase, Cochrane, and Web of Science were screened without time restriction up to December 12, 2025. The PubMed search strategy was as follows: ((((((“Appendicitis”[Mesh]) OR (Ruptured Appendicitis[Title/Abstract])) OR (Appendicitis, Ruptured)) OR (Perforated Appendicitis)) OR (Appendicitis, Perforated)) AND ((Child[Mesh]) OR (Children[Title/Abstract]))) AND ((“Interleukin-6”[Mesh]) OR (Interleukin 6 OR IL6 OR B-Cell Stimulatory Factor 2 OR B-Cell Stimulatory Factor-2 OR Differentiation Factor-2, B-Cell OR Differentiation Factor 2, B Cell OR B-Cell Differentiation Factor-2 OR B Cell Differentiation Factor 2 OR BSF-2 OR Hybridoma Growth Factor OR Growth Factor, Hybridoma OR IFN-beta 2 OR Plasmacytoma Growth Factor OR Growth Factor, Plasmacytoma OR Hepatocyte-Stimulating Factor OR Hepatocyte Stimulating Factor OR MGI-2 OR Myeloid Differentiation-Inducing Protein OR Differentiation-Inducing Protein, Myeloid OR Myeloid Differentiation Inducing Protein OR B-Cell Differentiation Factor OR B Cell Differentiation Factor OR Differentiation Factor, B-Cell OR Differentiation Factor, B Cell OR IL-6 OR Interferon beta-2 OR Interferon beta 2 OR beta-2, Interferon OR B Cell Stimulatory Factor-2 OR B Cell Stimulatory Factor 2[Title/Abstract])). Similar search strategies utilizing database-specific syntax were implemented for Embase, Cochrane, and Web of Science to ensure comprehensive coverage of the relevant literature.

### 2.2 Eligibility criteria

The following eligibility criteria were used: 1) children diagnosed with acute appendicitis were evaluated with concurrent measurement of IL-6 levels. 2) The study reported either the difference in IL-6 levels between acute appendicitis and non-appendicitis cases, or the difference between CA and UA.

### 2.3 Exclusion criteria

The exclusion criteria encompassed the following publication types: case reports, cross-sectional studies, reviews, books, letters to the editor, guidelines, dissertations, and commentaries. Furthermore, studies were excluded if they lacked essential data and could not be obtained upon request from the corresponding authors.

### 2.4 Data extraction

Two researchers independently conducted full-text screening, and inter-rater reliability was assessed using Cohen’s kappa coefficient. The kappa value for the full-text screening phase was 0.81, indicating excellent agreement between researchers. All discrepancies were resolved through discussion and consensus. The following information was extracted from each included study: first author, publication year, country, sample size (number of controls, UA, and CA), mean age, inflammatory markers, study design, and IL-6 levels. During data extraction, the specific IL-6 assay method [e.g., enzyme-linked immunosorbent assay (ELISA) and chemiluminescent immunoassay] and sample type (serum or plasma) for each included study were documented. For studies that did not explicitly report the assay method or kit source, an attempt was made to contact the corresponding author via email to obtain the missing information. If no response was received, the assay method was recorded as “not reported”. All data collection events were performed independently by two researchers, and any disagreements were resolved through discussion.

### 2.5 Quality of assessment

The methodological quality or risk of bias of the involved studies was assessed using the Newcastle–Ottawa scale (NOS). There were three domains to evaluate the quality of articles: the selection of the study participants, the comparability of the groups, and the assessment of outcome measures. Studies were classified according to quality: high quality (7–9), moderate quality (5–6), and poor quality (0–4).

### 2.6 Statistical analysis

Statistical analysis was performed using the R software 4.5.1 (R Foundation for Statistical Computing, Vienna, Austria). The “metafor” function in R was used for statistical analysis. Two reviewers independently extracted the data. The random-effects model was used, as it assumes varying effect sizes between included studies. The mean IL-6 level was calculated for each group and comparison. The mean difference (MD) was also calculated for research. Results were reported in a forest plot with 95% confidence intervals (CIs). The heterogeneity test applied in the study utilized the Q-test format and calculated the I^2^ statistic. I^2^ was quantified using the following guide: 0%–50% may represent low heterogeneity, 50%–75% may represent moderate heterogeneity, and 75%–100% may represent high heterogeneity. A funnel plot was performed to examine publication bias if there were at least 10 involved studies. To assess the independent predictive value of IL-6, the odds ratios (ORs) and 95% CI reported in the articles were processed and converted into log OR values for meta-analysis.

## 3 Result

### 3.1 Results of searches

**Figure 1:**
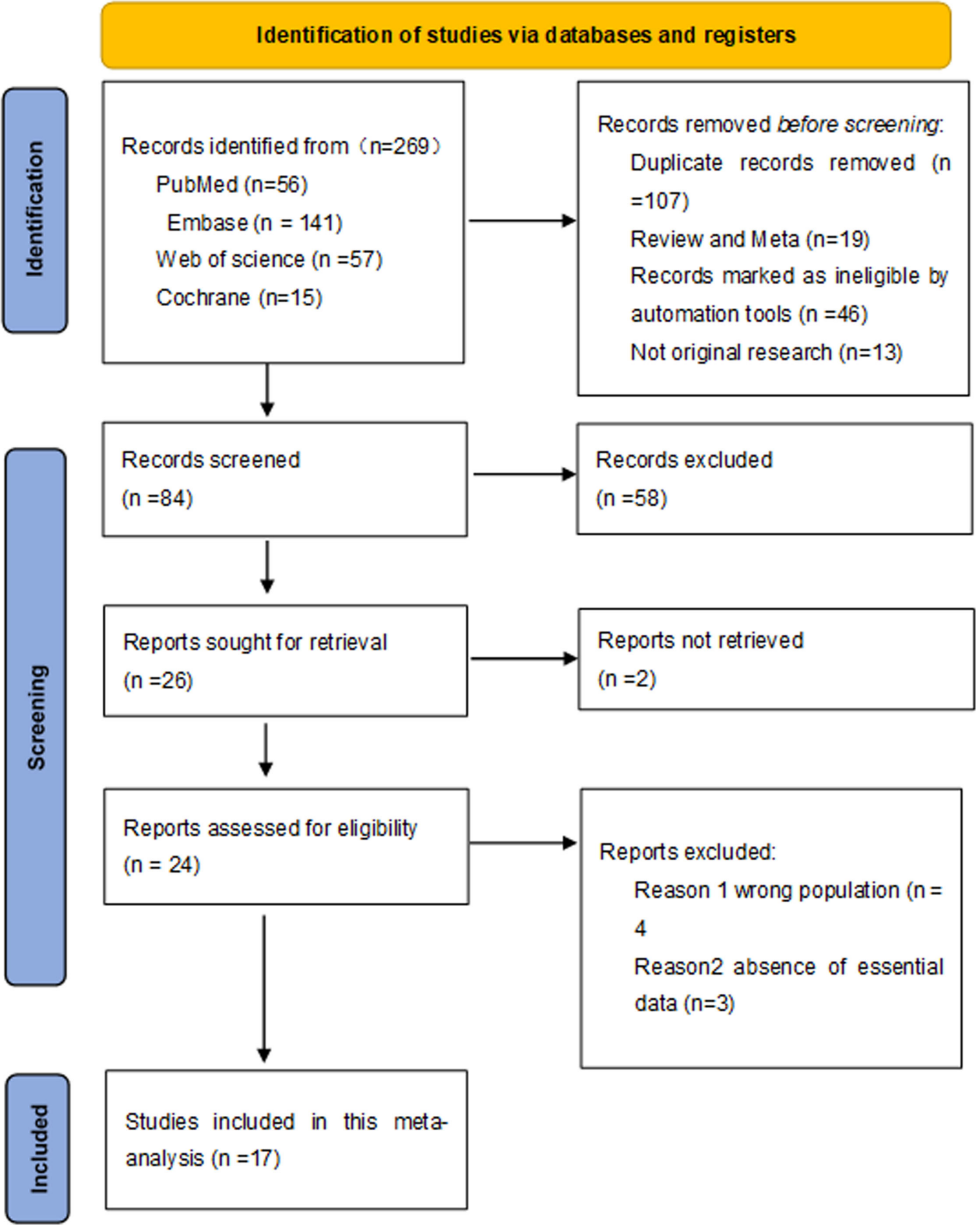
PRISMA flow diagram. PRISMA flow diagram delineates the systematic process of identifying and screening studies across multiple databases, culminating in the selection of 17 pertinent studies.

Searches of electronic databases identified 269 articles. Following the removal of duplicates, 84 studies underwent preliminary screening based on title and abstract. Of this, there were 17 studies that met the eligibility criteria and were included in this meta-analysis (**Figure 1**) (14–30). These studies enrolled a total of 3,104 patients. Seven studies (15, 20, 23–26, 28) were included in the comparison of appendicitis versus no appendicitis. A total of 283 patients were included in the no appendicitis group, and 392 patients were included in the appendicitis group. Ten studies (16–22, 25–27) were included in the comparison of UA versus CA. A total of 520 patients had UA, and 511 patients had CA. The majority of the studies were from Western countries, and the population characteristics are shown in **Tables 1**, **2**.

**Table 1:** Characteristics of the studies included in the meta-analysis.

Author	Year	Country	Design	Included population	Sample size	Mean age (year)	Newcastle–Ottawa scale score
Martinez	2025	Spain	Prospective observational case–control study	Abdominal pain due to appendicitis and children with abdominal pain from other causes	160	NR	7
Li X	2025	China	Retrospective cohort study	Pathologically confirmed acute appendicitis and healthy controls	300	11	6
Zhou J	2025	China	Retrospective analysis	Post-surgery pathological results	483	NR	9
Lin WY	2024	China	Retrospective cohort study	Pathologically confirmed acute appendicitis	226	10	8
Elliver	2024	Sweden	Prospective cohort study	Children confirmed appendicitis	177	NR	8
Zhang T	2023	China	Prospective cohort study	Pathologically confirmed acute appendicitis	140	6.93	6
Sahin, C.	2023	Germany	Prospective and controlled study	Pathologically confirmed acute appendicitis and healthy controls	138	12	7
Di Mitri, M.	2022	Italy	Prospective observational study	Not inflamed appendix, not complicated appendix, and complicated appendix	228	10.3	8
Arredondo M	2022	Spain	Prospective observational study	Pathologically confirmed acute appendicitis	205	NR	9
Yongkang X	2021	China	Prospective observational study	Pathologically confirmed acute appendicitis	96	5.5	8
Lin Z	2021	China	Prospective observational study	Pathologically confirmed acute appendicitis	307	NR	7
Kakar	2020	Latvia	Prospective single-center cohort study	Control, acute complicated appendicitis, and acute uncomplicated appendicitis patients	92	12.3	8
Zviedre	2016	Latvia	Prospective case–control study	Acute appendicitis and acute mesenteric lymphadenitis	74	NR	6
Ozguner	2014	Turkey	Prospective controlled trial	Non-complicated appendicitis, complicated appendicitis, and non-specific abdominal pain	49	9.9	7
Huang D	2012	China	Prospective observational study	Pathologically confirmed acute appendicitis	67	7.2	6
Kharbanda	2011	USA	Prospective cohort study	Final diagnosis was determined by histopathology	280	11.3	7
Groselj-Grenc	2007	Slovenia	Prospective study	Pathologically confirmed acute appendicitis	82	NR	7

**Table 2:** Characteristics of the studies included in the meta-analysis.

Author	Sample size	No appendicitis	Appendicitis	Uncomplicated appendicitis	Complicated appendicitis
Martinez	160	80	80	NR	NR
Li, X.	300	150	150	NR	NR
Zhou, J.	483	0	483	207	276
Lin, W.Y.	226	NR	NR	106	102
Elliver	177	40	137	79	58
Zhang, T.	140	0	140	97	43
Sahin, C.	138	73	65	33	32
Di Mitri, M.	228	23	84	63	21
Arredondo, M.	205	57	95	65	30
Yongkang, X.	96	0	96	30	66
Lin, Z.	307	0	307	83	224
Kakar	92	29	63	31	32
Zviedre	74	17	31	NR	NR
Ozguner	49	15	34	17	17
Huang, D.	67	16	51	19	22
Kharbanda	280	186	94	72	22
Groselj-Grenc	82	33	49	NR	NR

All included studies were conducted in pediatric populations. However, the reporting of age data was incomplete in a subset of studies; specifically, seven studies (14, 17, 22, 24, 28–30) did not provide detailed mean age data, which we have recorded as “not reported” (NR) in our data extraction (**Table 1**).

All diagnostic samples were serum samples. Of the 17 studies included in the review, six used ELISA (21–23, 25–27). Among the remaining 11 studies, three employed chemiluminescence (16, 17, 30), one utilized Luminex xMAP technology (24), and one used a chemiluminescent immunoassay (28). The other six studies (14, 15, 18–20, 29) did not specify the detection method used. Consequently, these 11 studies were all classified under the “other” detection methods category.

### 3.2 Quality and evidence assessment

The Newcastle–Ottawa scale (NOS) was used to assess the methodological quality or bias risk of the involved studies. Thirteen studies showed low risk of bias among 17 studies, and four studies showed moderate quality (**Table 1**). The main biases were within the comparability domain for studies that did not include multivariate analyses and for the representativeness of the exposed cohort.

### 3.3 Pooled analysis of no appendicitis versus appendicitis

**Figure 2:**
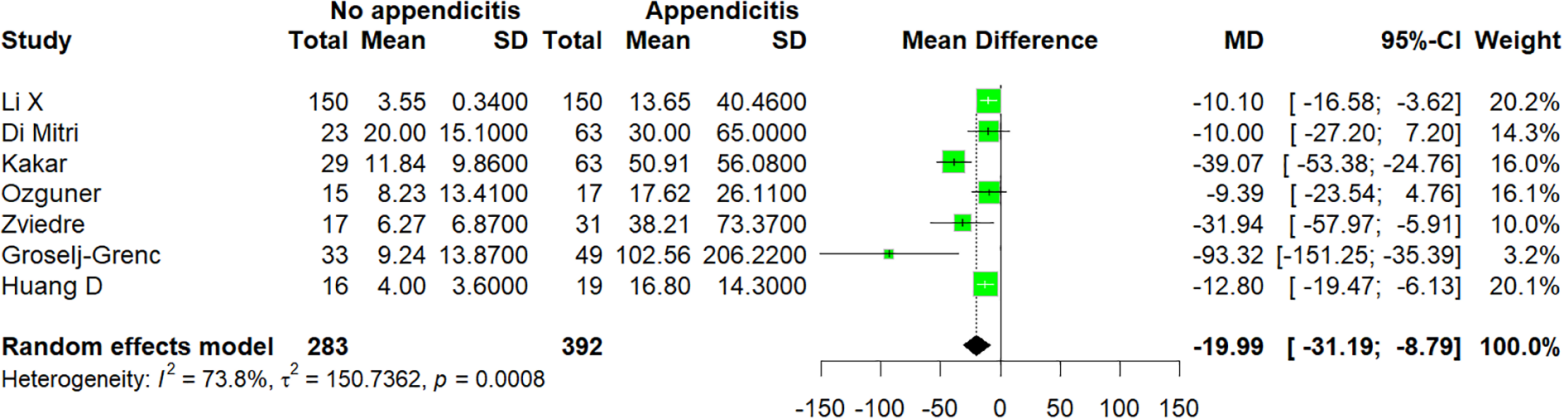
Forest plot comparing IL-6: no appendicitis versus appendicitis.

Seven studies (15–18, 20, 23, 28) were included in this comparison, which enrolled a total of 675 patients. There was a statistically significant difference between the appendicitis group and the no appendicitis group (MD −19.99; 95% CI −31.19 to −8.79; p = 0.0008; I^2^ = 73.8%; **Figure 2**). A moderate level of heterogeneity among the studies existed (I^2^ = 73.8%; p = 0.0008). This indicator was excluded from publication bias analysis due to fewer than 10 included studies.

### 3.4 Pooled analysis of uncomplicated versus complicated appendicitis

**Figure 3:**
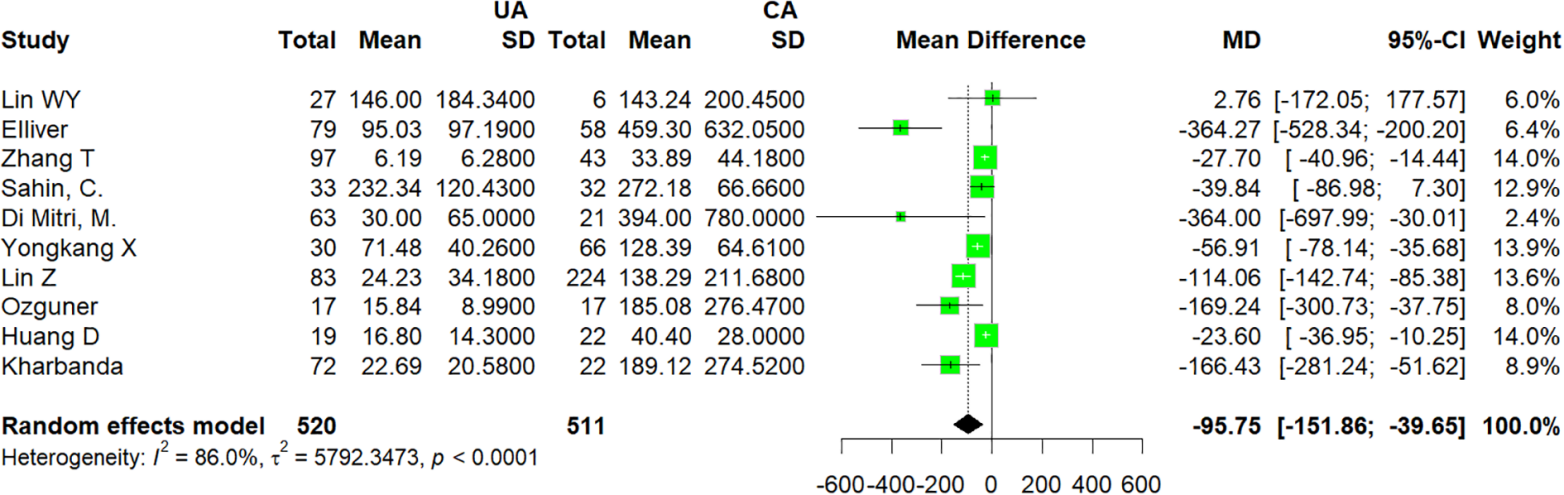
Forest plot comparing IL-6: uncomplicated appendicitis versus complicated appendicitis. UA, uncomplicated appendicitis; CA, complicated appendicitis.

**Figure 4:**
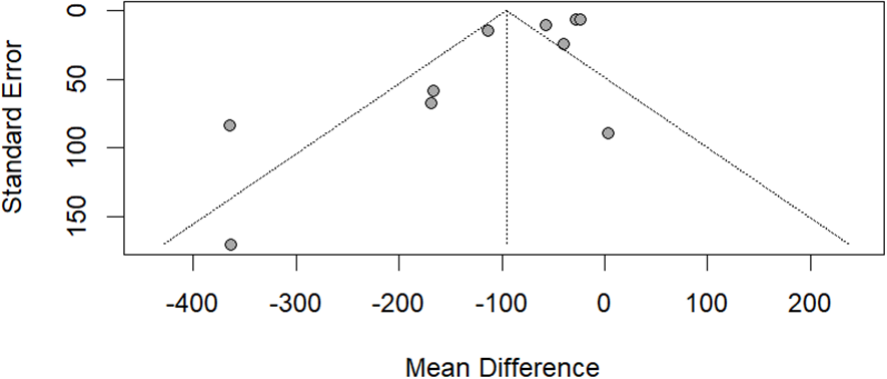
Funnel plot of the comparisons of IL-6: uncomplicated appendicitis versus complicated appendicitis.

Ten studies (16–22, 25–27), which enrolled 1041 patients, compared IL-6 between UA and CA. There was a statistically significant difference between the UA group and CA group (MD −95.75; 95% CI −151.86 to −39.65; p < 0.0001; I^2^ = 86%; **Figure 3**). A high level of heterogeneity among the studies existed (I^2^ = 86%; p < 0.0001). The likelihood of publication bias was low based on the funnel plot (**Figure 4**).

### 3.5 Subgroup analysis

We conducted a subgroup analysis based on the methods used to detect IL-6. ELISA was the primary detection method, while other methods included chemiluminescent immunoassay, Luminex technology, and studies employing unspecified detection methods. We divided the studies into two groups: the ELISA group (using enzyme-linked immunosorbent assay) and the other methods group (including chemiluminescent immunoassay, Luminex technology, and studies using unspecified detection methods). Only five studies assessed the independent predictive value of IL-6. Subgroup analysis based on detection method was not performed for complicated appendicitis, as this would have resulted in subgroups containing fewer than three studies.

**Figure 5:**
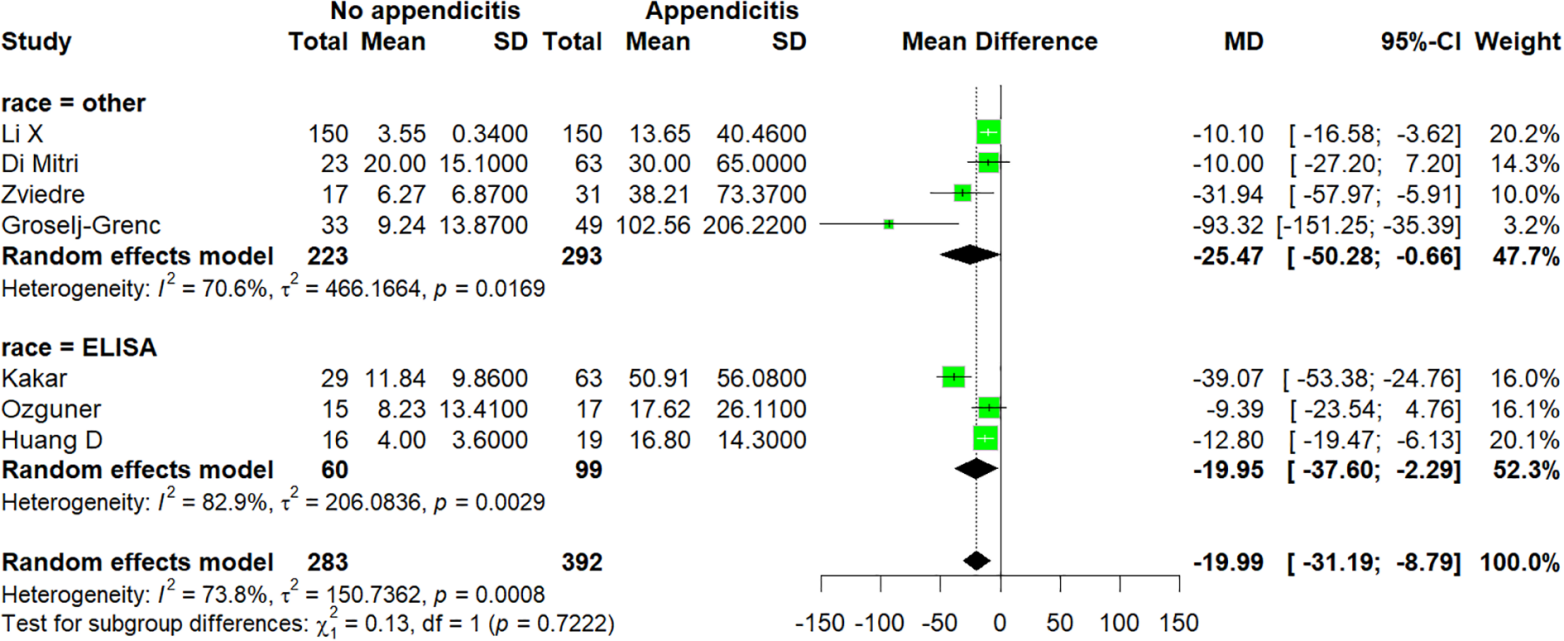
Subgroup forest plot comparing IL-6: no appendicitis versus appendicitis.

Subgroup analysis of studies comparing IL-6 levels between non-appendicitis and appendicitis patients revealed that in the “ELISA” subgroup comprising three studies, the pooled MD was −19.95 (95% CI: −37.60 to −2.29), with high heterogeneity (I^2^ = 82.9%, p = 0.0029). In the “other” subgroup, comprising four studies, the pooled MD was −25.47 (95% CI: −50.28 to −0.66), with moderate heterogeneity between studies (I^2^ = 70.6%, p = 0.0169); the overall random-effects model indicated heterogeneity among the studies (I^2^ = 73.8%, p = 0.0008). Differences between subgroups were not statistically significant (χ^2^ = 0.13, p = 0.7222), suggesting that subgroup analysis had no significant impact on the effect size (**Figure 5**).

**Figure 6:**
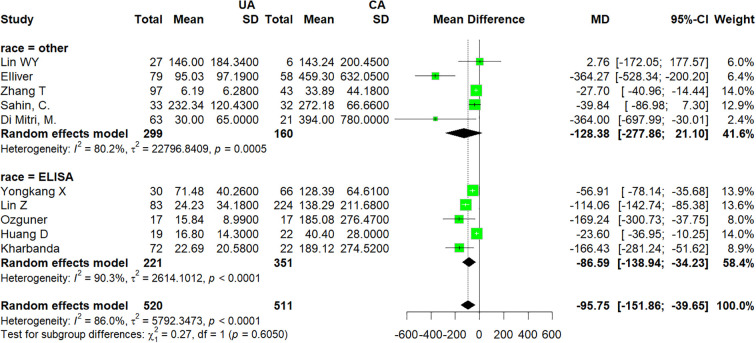
Subgroup forest plots of the comparisons of IL-6: uncomplicated appendicitis versus complicated appendicitis. UA, uncomplicated appendicitis; CA, complicated appendicitis.

Subgroup analyses of studies on the UA group and CA group revealed that in the “ELISA” subgroup, which included five studies, the pooled effect size was MD = −86.59 (95% CI: −138.94 to −34.23), with high heterogeneity (I^2^ = 90.3%, p < 0.0001). In the “other” subgroup, a total of five studies were included, with a pooled effect size of MD = −128.38 (95% CI: −277.86 to 21.10), and there was high heterogeneity among the studies (I^2^ = 80.2%, p = 0.0005). The overall random-effects model, combining the 10 studies, yielded a meta-analysis effect size of MD = −128.38 (95% CI: −277.86 to 21.10), with significant heterogeneity (I^2^ = 86.0%, p < 0.0001). The test for differences between subgroups was not statistically significant (χ^2^ = 0.27, p = 0.6050), suggesting that there were no significant differences in effect sizes between the different subgroups (**Figure 6**).

### 3.6 Sensitivity analysis

**Figure 7:**
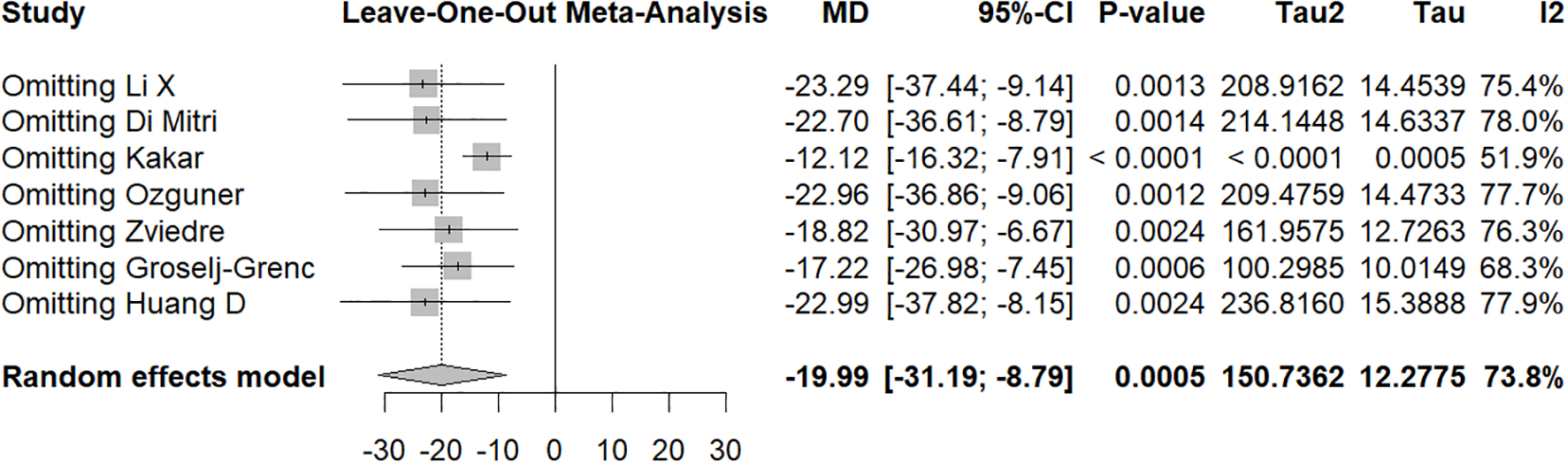
Leave−one−out sensitivity analysis plot for studies comparing non−appendicitis versus appendicitis.

**Figure 8:**
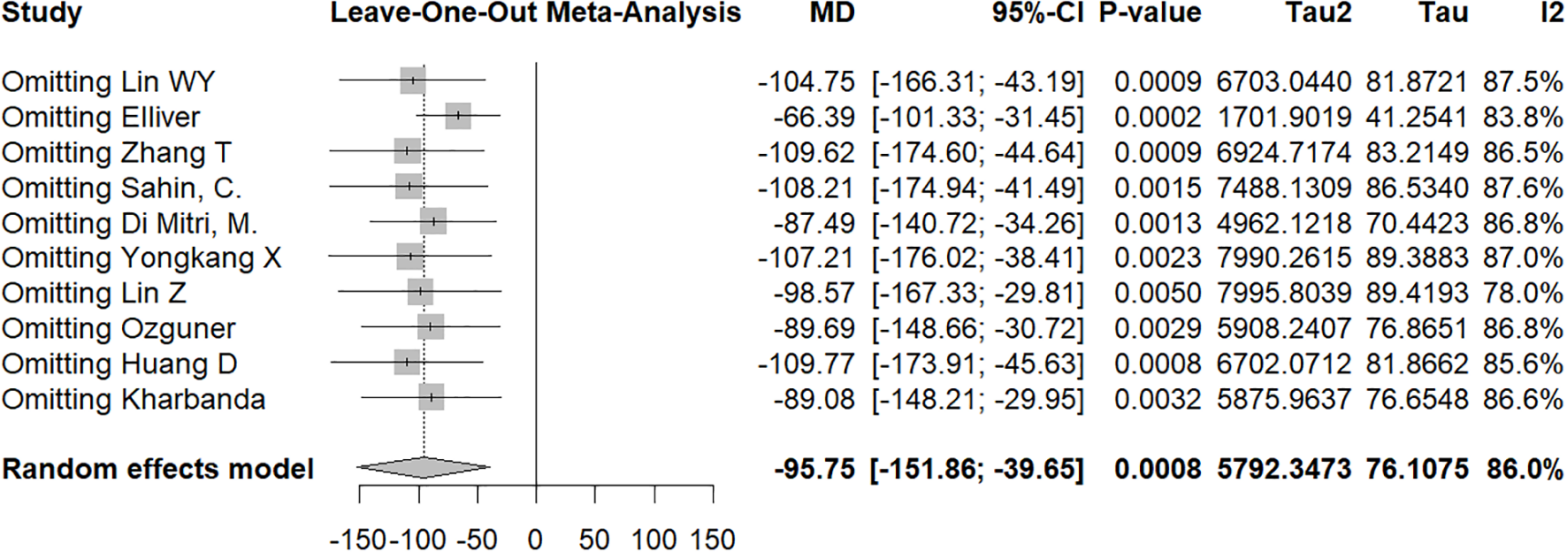
Leave−one−out sensitivity analysis plot for studies comparing non−complicated appendicitis versus complicated appendicitis.

In the first sensitivity analysis, the pooled MD was −19.99 (95% CI: −31.19 to −8.79, p = 0.0005), with moderate heterogeneity (I^2^ = 73.8%) (**Figure 7**). After excluding the study by Kakar et al., heterogeneity decreased significantly (I^2^ = 51.9%), and the effect size became −12.12 (95% CI: −16.32 to −7.91), suggesting that this study may have been one of the main sources of heterogeneity; importantly, this exclusion did not materially alter the direction or significance of the overall conclusion. In the second subgroup analysis, the overall pooled MD was −95.75 (95% CI: −151.86 to −39.65, p = 0.0008), with high heterogeneity (I^2^ = 86.0%) (**Figure 8**). Leave-one-out analysis demonstrated that no single study excessively influenced the pooled estimate; the MD ranged from −66.39 to −109.77, all of which remained statistically significant.

### 3.7 Analysis of the independent predictive value of IL-6 in complicated appendicitis

**Figure 9:**
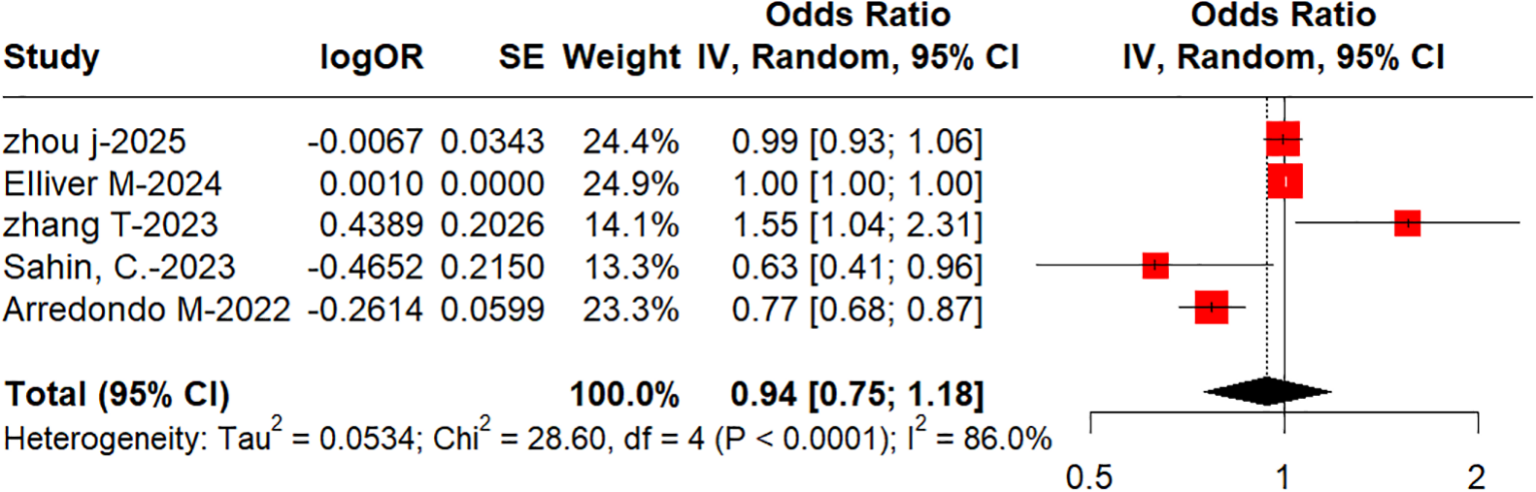
Forest plot of the independent predictive value of IL-6 in complicated appendicitis.

Five studies (17–19, 29, 30), which involved 1,143 patients, evaluated the independent predictive value of IL-6 in CA (**Figure 9**). The pooled odds ratio (OR) was 0.94 (95% CI: 0.75–1.18), with the interval encompassing 1 (the null line). This indicates that the association lacks statistical significance, while the pooled effect size test demonstrated high statistical significance (p < 0.0001). A high level of heterogeneity among the studies existed (I^2^ = 86%; p < 0.0001).

## 4 Discussion

This systematic review analyzed and compared IL-6 levels in patients with and without appendicitis, as well as in cases of CA, to evaluate whether this marker can distinguish CA. This meta-analysis identified 17 studies, enrolling a total of 3,104 patients. All included studies were conducted in pediatric populations and used serum samples. The detection methods employed included ELISA, chemiluminescence, and other techniques. The principal findings can be summarized in three key points: first, IL-6 demonstrates good diagnostic accuracy in distinguishing children with appendicitis from those without appendicitis. Second, IL-6 levels are significantly higher in children with CA compared to those with UA. Third, multivariate analyses from the included studies suggest that IL-6 cannot be an independent predictor for CA. The quality of the available evidence was moderate to high, and the results remained consistent through sensitivity analyses and separate analyses.

This is the first study to incorporate IL-6 as a biomarker and investigate whether there is a significant difference in IL-6 levels between UA and CA. IL-6 is a multifunctional inflammatory cytokine whose production is primarily induced by upstream pro-inflammatory mediators, namely, tumor necrosis factor-alpha (TNF-α) and interleukin-1 beta (IL-1β) (31). IL-6, a classic pro-inflammatory cytokine, exacerbates local tissue damage and systemic inflammation by promoting inflammatory cascades, thereby increasing overall disease severity (32). In the pathological process of acute appendicitis, obstruction of the appendiceal lumen leads to bacterial overgrowth, local tissue ischemia, and massive release of inflammatory mediators. Our analysis confirms that IL-6 concentrations are significantly higher in children with CA than in those with UA. This finding is consistent with the pathophysiological mechanism of CA, where appendiceal perforation or gangrene leads to intra-abdominal infection and a more intense systemic inflammatory response, thereby stimulating the production of higher levels of IL-6. Therefore, IL-6 detection not only aids in identifying the presence of appendicitis but also reflects the severity of inflammation, providing an objective laboratory parameter for assessing disease status.

We note the heterogeneity in the existing evidence and tentatively suggest that it may stem from differences in the methods used to detect IL-6 across the various studies. Although the heterogeneity in the ELISA group (I^2^ > 90%) was higher than that in the “other” group, the difference between subgroups was not statistically significant. This suggests that the heterogeneity may not be caused by the testing method itself but is more likely related to other factors within the individual studies. The possible reasons for the higher heterogeneity in the ELISA group include the clinical diversity of the included studies: factors such as patient age, duration of illness, and severity of appendicitis may vary considerably across different studies; as the immune system matures during childhood, IL-6 levels may vary according to developmental stage. This is one of the reasons why there may be heterogeneity in the age factor, or differences in testing procedures: even when using the same ELISA method, there may be variations in the reagents, antibodies, and threshold settings employed in different studies. Therefore, although the heterogeneity values are higher in the ELISA group, this cannot be simply attributed to the testing method itself. It is recommended that future studies further standardize the ELISA testing procedures and validate their diagnostic performance in larger sample sizes. Although univariate analysis revealed significant differences in IL-6 levels between appendicitis and non-appendicitis, as well as between UA and CA, IL-6 did not emerge as an independent predictor of CA in the multivariate regression model (pooled OR = 0.94, 95% CI: 0.75–1.18, p > 0.05). This contradictory finding may be due to the following reasons: as an acute-phase inflammatory mediator, IL-6 is highly correlated with traditional inflammatory markers such as CRP, white blood cell count, and neutrophil percentage. In multivariate analysis, when these markers are included simultaneously, the independent effect of IL-6 may be weakened or masked. Although IL-6 is associated with appendicitis and its severity in univariate analysis, its inability to serve as an independent predictor of CA is attributed to its multicollinearity with other inflammatory markers, high heterogeneity across studies, and the adjustment for confounding factors in the multivariate model. Future large-scale, prospective, multicenter studies are required to standardize detection methods and cut-off values and to develop predictive models in order to clarify the clinical value of IL-6.

The findings of this study hold clear clinical significance. At the diagnostic level, for children with abdominal pain exhibiting atypical clinical presentations and diagnostic challenges, IL-6 testing can serve as a valuable supplement to existing clinical evaluation systems, particularly for those with negative ultrasound findings. This holds promise for further improving diagnostic accuracy and reducing the risk of perforation due to delayed diagnosis or misdiagnosis. Regarding treatment decisions, elevated preoperative IL-6 levels can alert surgeons to the risk of CA, aiding in more precise planning of surgical timing, selection of surgical approach, and thorough preoperative counseling. Additionally, this helps identify patients with mild clinical symptoms but high-risk biomarker profiles, thereby avoiding unnecessary observation and waiting. From a pediatric care perspective, a reliable biomarker could reduce reliance on CT scans, lower radiation exposure risks for children, and potentially decrease the rate of negative appendectomies.

We must objectively acknowledge the limitations of this meta-analysis. First, most included studies employed retrospective designs, carrying inherent risks of selection bias and information bias. Second, significant heterogeneity indicates methodological differences across studies. Although we explored some sources through subgroup analyses and sensitivity analyses, we could not fully eliminate their impact, limiting our ability to recommend a unified diagnostic cutoff value. Third, evidence regarding IL-6 as an independent predictor primarily stems from multivariate analyses in a limited number of studies, requiring validation through additional prospective research to establish its generalizability. Finally, IL-6 testing is not yet a routine procedure in all healthcare settings. Practical challenges—including testing costs, turnaround time, and the establishment of pediatric-specific reference ranges—must be addressed before widespread clinical adoption can occur.

## 5 Conclusion

IL-6 is a promising marker that can predict both the diagnosis and severity of appendicitis. IL-6 levels are significantly elevated in pediatric appendicitis patients, with more pronounced increases observed in CA. Our systematic review indicates that IL-6 may serve as one of the markers for distinguishing UA from CA, although no clear association was found regarding its independent predictive value. Future prospective studies with larger sample sizes and standardized detection methods are warranted to establish higher-level evidence.

## Data Availability

The original contributions presented in the study are included in the article/Supplementary Material. Further inquiries can be directed to the corresponding author.
